# A Tale of Two Immune Cells in Rheumatoid Arthritis: The Crosstalk Between Macrophages and T Cells in the Synovium

**DOI:** 10.3389/fimmu.2021.655477

**Published:** 2021-06-17

**Authors:** Jiajie Tu, Wei Huang, Weiwei Zhang, Jiawei Mei, Chen Zhu

**Affiliations:** ^1^ Institute of Clinical Pharmacology, Anhui Medical University, Key Laboratory of Anti-Inflammatory and Immune Medicine, Ministry of Education, Anhui Collaborative Innovation Center of Anti-Inflammatory and Immune Medicine, Hefei, China; ^2^ Department of Gynecology, The First Affiliated Hospital of Shenzhen University, Health Science Center, Shenzhen Second People’s Hospital, Shenzhen, China; ^3^ Department of Orthopaedics, The First Affiliated Hospital of University of Science and Technology of China (USTC), Division of Life Sciences and Medicine, University of Science and Technology of China, Hefei, China; ^4^ Departments of Geriatrics, The First Affiliated Hospital of USTC, Division of Life Sciences and Medicine, University of Science and Technology of China, Hefei, China

**Keywords:** RA, T cells, macrophage, pathogenesis, targeted therapy

## Abstract

Rheumatoid arthritis (RA) is a chronic inflammatory autoimmune disease. Joint inflammation of RA is closely related to infiltration of immune cells, synovium hyperplasia, and superfluous secretion of proinflammatory cytokines, which lead to cartilage degradation and bone erosion. The joint synovium of RA patients contains a variety of immune cellular types, among which monocytes/macrophages and T cells are two essential cellular components. Monocytes/macrophages can recruit and promote the differentiation of T cells into inflammatory phenotypes in RA synovium. Similarly, different subtypes of T cells can recruit monocytes/macrophages and promote osteoblast differentiation and production of inflammatory cytokines. In this review, we will discuss how T cell-monocyte/macrophage interactions promote the development of RA, which will provide new perspectives on RA pathogenesis and the development of targeted therapy.

## Introduction

Rheumatoid arthritis (RA) is a chronic autoimmune disease that seriously affects human health. A variety of immune cells are involved in the pathogenesis of RA (1), including cells from the innate immune system, such as macrophages, dendritic cells (DCs), and natural killer (NK) cells; and from the adaptive immune system, such as T lymphocytes (T cells) and B lymphocytes (B cells). In addition, some non-immune cells, fibroblasts, and endothelial cells are also involved in the development of RA. The interaction among these cellular components in joint synovium is quite complicated, including T cells and DC cells (2), T cells and NK cells (3), macrophages and fibroblasts (4), etc. Among them, T cells (5) and macrophages (6) are recognized as two critical cellular components involved in RA.

The essential role of T cells in the pathogenesis of RA has been validated, including studies on the infiltration of synovial T cells in inflammatory synovium of RA (7). However, the specific effects of T cells subsets and related cytokines on other immune cells in RA is elusive. Furthermore, it is also uncertain how other cellular components (such as macrophages) in modulate the activation, polarization and function of subpopulations of CD4^+^ T cells in joint synovium of RA. On the other hand, macrophages are also important in the development of RA (8). A series of studies have found that the heterogeneity of the synovial macrophages is quite high (9–11), and synovial macrophages are modulated by direct contact (cell-cell interaction) or indirect regulation (by cytokines produced by other cells, such as T cells, B cells and fibroblasts) in RA synovium (8). The ratio of inflammatory(M1) and anti-inflammatory(M2) macrophages is impaired in RA (9). CD14^+^ Bone marrow (BM) monocytes/macrophages are present in the joint synovium of RA patients, and they produce co-stimulatory molecules and inflammatory cytokines, and present an active phenotype (12, 13). In RA synovial fluid, the frequency of CD14^++/bright^ CD16^+^ monocyte population increase compared to that of healthy controls (14). After treatment with sodium aurothiomalate (SAT), a widely-used disease modifying drugs (DMARDs), the CD68^+^ macrophages around blood vessels and connective tissue area decreased in synovium of RA patients (14); furthermore, a significant correlation between lower macrophage counts and favorable radiological results was observed in these patients. In addition, it has been reported that a decrease in the synovial CD68^+^ macrophages amount was significantly associated with clinical improvement (15). Currently, a series of drugs that target macrophage-related factors are in clinical trial (16).

Although the functions of monocytes/macrophages and T cells in RA have been investigated for many years, the study of their interactions in RA has been scarcely approached. Colocalization of monocytes and T cells has been observed in RA synovium (17), implying that T cell-monocyte/macrophage interactions may occur at the site of inflammation. Given the critical role of T cells and macrophages in RA, their interaction could be an essential factor to consider as it may also play a central role in the development of this autoimmune pathology (18). Therefore, to illustrate the specific interaction between T cells and macrophages is essential to understand the molecular pathogenesis of RA. This mini-review summarizes previous research articles on T cell-monocyte/macrophage interactions in RA, highlighting the key role of the “crosstalk” between these cells in RA and pointing out possible directions for future studies.

## Macrophages Regulate T Cells in RA

**Figure 1:**
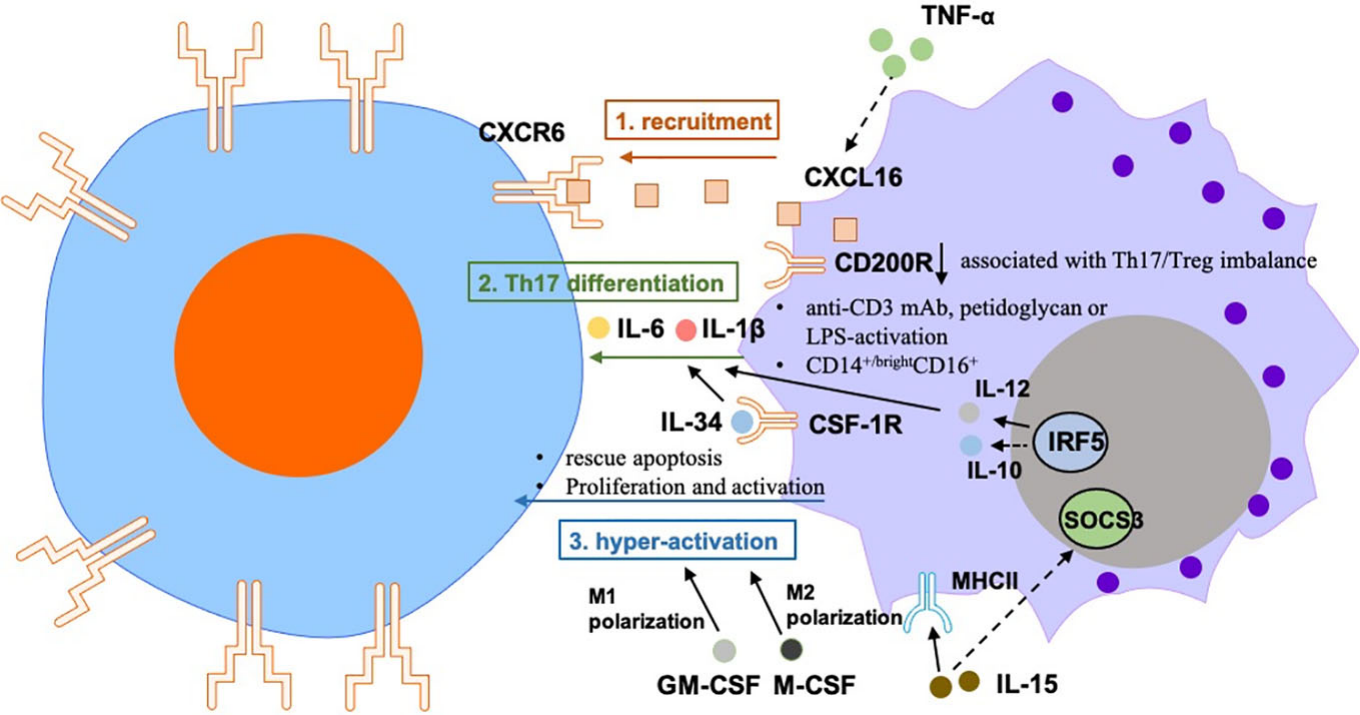
The regulation of T cells by macrophages in RA is mainly reflected on the 1) recruitment: Macrophages-secreted CXCL16 induces migration of CXCR6^+^ T cells in RA joint synovium, which is regulated by TNF-α. 2) Th17 differentiation: abnormal expression of CD200R1 was associated with Th17/Treg imbalance in patients with active RA. Treatment with anti-CD3 mAb, peptidoglycan, or LPS-activated monocytes from peripheral blood can induce IL-17 secretion from CD4^+^ T cells. Treatment with anti-CD3/CD28-activated CD4^+^ T cells can boost Th17 polarization of PBMCs that were treated with RA synovial fluid from healthy donors, which may be due to the up-regulation of IL-6 and IL-1β from monocytes. Human CD14^+/bright^ CD16^+^ monocytes promoted Th17 differentiation of memory CD4^+^ T cells. Activation of the IL-34-CSF-1R pathway in synovial macrophages can promote Th17 differentiation of T cells. IRF5 promotes monocytes/macrophages-induced Th17 differentiation of T cells. 3) induction of hyper-activation of T cells by monocytes/macrophages: IL-15 ﻿lead to increased expression of MHCII and ﻿reduced expression of the SOCS3 in macrophages, which activate the proliferation of autoreactive CD4^+^ T cells in RA. Monocytes rescue synovial T cells from glucocorticoid-induced apoptosis. LPS + IFNγ-treated M-CSF-dependent macrophages inhibit the proliferation, activation and cytokine production of CD4^+^ T cells.

The regulation of T cells by macrophages in RA is mainly reflected on the activation and amplification of T cells, and subsequent T  cell priming by monocytes/macrophages (**Figure 1**).

### Macrophages Recruit T Cells in RA

Monocytes/macrophages recruit and maintain homeostasis of CD4^+^ T cells in synovium from RA patients (17, 19). C-X-C motif chemokine receptor (CXCR6) highly expressed in type 1 polarized effector memory T cells in synovial fluid from RA patients (20). It has been reported that the expression of CXCR6 in T cells in joint synovium of RA patients was consistent with the upregulation of CXCL16 (the ligand of CXCR6) in synovial CD14^+^ monocytes/macrophages (20, 21). *In vitro* migration experiments demonstrated that CXCL16 induces migration of CXCR6^+^ T cells isolated from RA patient’s joint synovium (22).

CXCL16 is regulated by two groups of cytokines: Th2-related cytokines IL-4 and IL-10, which inhibit the secretion of CXCL16 in monocytes/macrophages from RA patients; and Th1-related cytokine IFN, which enhances CXCL16 secretion (23). Moreover, earlier studies have found that TNF-α-treated human monocytes promote transmembrane expression of CXCL16, suggesting that the synovial TNF-α may affect the recruitment of CXCR6^+^ T cells (20).

In a study that included three patients who received anti-TNF-α therapy, the *in situ* immunohistochemistry results showed a significant reduction of CXCL16 in the synovium. This observation may be due to a reduction in the number of monocytes in joint synovium after treatment, as it is known that synovial cellularity rapidly decreases after anti-TNF-α therapy (24). In contrast, CXCL16 expression remained high in three patients who did not respond to anti-TNF-α therapy. The expression of CXCL16 decreased in both the joint synovium and serum of patients who responded to the TNF treatment (25). These data suggest that upregulation of CXCL16 in macrophages/monocytes promotes the recruitment of CXCR6^+^ T cells in RA joint synovium, which may help understand the pathological mechanisms of synovitis. However, the effect of CXCL16 is not specific to monocytes/macrophages in RA. Other antigen presenting cells, such as B cells (1) and DC cells (2), also are potential sources of CXCL16 in RA.

### Macrophages Promote Th17 Differentiation in RA

It was observed that the expression levels of CD200R1 on macrophages of RA patient are lower than that of healthy controls. This abnormal expression was associated with Th17/Treg imbalance in patients with active RA (26). In addition, CD200R1 expression negatively correlated with DAS28, ESR, and CRP levels.

It has been shown that both murine and human monocytes/macrophages from arthritis joint synovial fluid can promote the production of IL-17 in CD4^+^ T cells (27–29). In accordance, treatment with anti-CD3 mAb, peptidoglycan, or LPS-activated monocytes from peripheral blood can effectively induce IL-17 secretion from human CD4^+^ T cells (30). Treatment with anti-CD3/CD28-activated CD4^+^ T cells can also boost Th17 polarization of PBMCs that were treated with RA synovial fluid from healthy donors, which may be due to an increase of the IL-6 and IL-1β produced by monocytes (31). Rossol et al. demonstrated that human CD14^+/bright^ CD16^+^ monocytes promoted Th17 differentiation of memory CD4^+^ T cells. The presence of CD14 ^+/bright^ CD16^+^ monocytes was positively correlated with Th17 cell density in PBMCs from RA patients (32). Accordingly, it has been reported that activation of the IL-34-CSF-1R pathway in peripheral monocytes can promote Th17 differentiation of T cells from RA patients. In this sense, in an *in vitro* co-culture experiment, binding of IL-34 to IL-34-CSF-1R promoted the secretion of IL-6 by THP-1 cells (human monocyte cell line) and increased percentage of Th17 cells through IL-6 production. It was also shown that ROS levels were induced in this co-culture model (33).

The expression of IRF5 in human macrophages can be reversibly induced by inflammatory stimulation and contributes to macrophage polarization (34). IRF5 is a marker of M1 macrophages, which  directly activates transcription of interleukin 12 subunit p40 (IL-12p40), IL-12p35, and IL-23p19; and represses IL-10. In addition, M1 macrophages prepare the micro-environment for a potent response of Th1/Th17. Transcriptome analysis has proven that exogenous IRF5 upregulates or downregulates M1 or M2 associated phenotypic markers, respectively (34). However, these studies only show that inflammatory monocytes/macrophages promote Th17 differentiation of T cells under certain conditions (mostly inflammatory stimulation *in vitro*), it is important to further illustrate how the specific mechanisms involved in T cell-monocyte/macrophage interactions could favor the development of novel targeted therapies.

### Macrophages Promote the Hyper-Activation of T Cells in RA

Besides producing inflammatory cytokines and chemokines, monocytes/macrophages also play a role in adaptive immune system, which involves the pathogenesis of RA (13). In RA synovium, CD14^+^ cells co-locate with CD4^+^ T cells, indicating that monocytes/macrophages and T cells may crosstalk *in vivo* in an inflammatory environment (17). Other related studies mainly focused on how macrophages promote the hyper-activation of T cells in RA.

Monocytes rescue synovial T cells from glucocorticoid-induced apoptosis, which is a specific feature of RA. ﻿Co-culture of monocytes and T cells from RA patients showed that soluble factors are important for T cell resistance to glucocorticoid-mediated apoptosis; however, the study does not clarify which cytokines secreted by macrophages inhibited T cells apoptosis caused by the glucocorticoids (35).

﻿Interleukin-15 (IL-15) is a proinflammatory cytokine that is overexpressed in RA. In this context, excessive amounts of IL-15 ﻿lead to increased expression of major histocompatibility complex class (MHC) II and ﻿reduced expression of the suppressor of cytokine signaling (SOCS) 3 in macrophages, which activate the proliferation of autoreactive CD4^+^ T cells in RA (36).

GM-CSF-stimulated macrophages demonstrate inflammatory feature, specific of M1 macrophages; while M-CSF-dependent macrophages show phenotype of M2 polarization. LPS + IFNγ-treated M-CSF-dependent macrophages inhibit the proliferation, activation and cytokine production of CD4^+^ T cells (37). Both human (CD14^+^CD68^+^) and murine (CD45^+^CD11b^+^GR-1^-^) inflammatory synovial macrophages can further amplify the hyper-activation of T cells (28, 38), which is an important cause of RA. Therefore, the key to treating RA is to interrupt the source of the amplification cascade.

## T Cells Modulate Macrophages in RA

**Figure 2:**
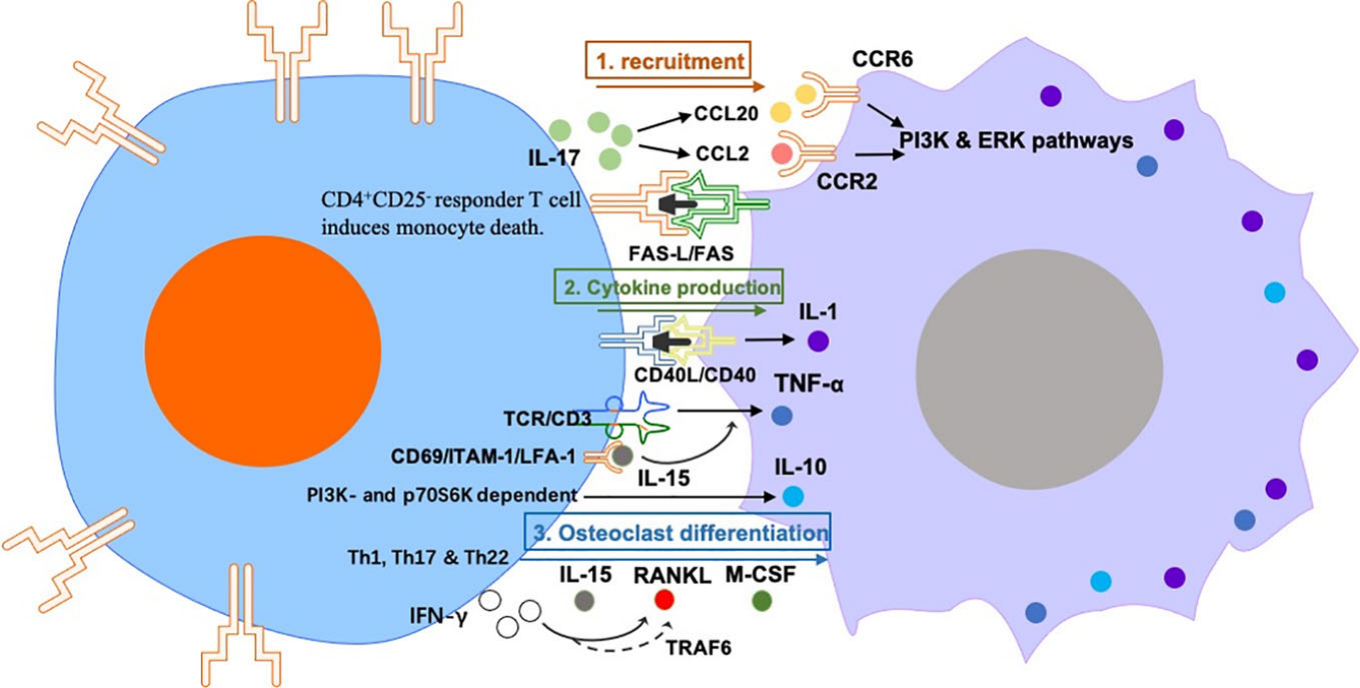
The regulation of macrophages by T cells in RA mainly includes effects on macrophage 1) recruitment: Synovial Th17 cells secrete CCL20 and CCL2, which has chemotactic effects on monocytes. ﻿The induction of monocyte death requires activation of CD4^+^ CD25^-^ responder T cell–cell contact in a FAS-L/FAS dependent manner. 2) cytokine production: anti-CD3 activated peripheral CD4^+^ T cells can activate monocytes to produce IL-1 in a CD40-CD40L dependent manner. TCR/CD3-mediated T cell activation induces monocyte TNF-α production, which is induced by IL-15. IL-10 production in RA synovial-membrane mononuclear cells and M-CSF-primed macrophages is activated by interaction with ﻿cytokine-stimulated T cells in a PI3K- and p70S6K-dependent manner. and 3) osteoclast differentiation: Th1, Th17 and Th22 can induce osteoclast differentiation *via* producing IL-15, RANKL, M-CSF and IFN-γ. However, IFNγ also disrupts the differentiation of osteoclasts *via* degrading RANK bridging protein TRAF6, this suggests that IFNγ^+^ T cells can promote or hinder osteoclastogenesis under different conditions.

The regulation of macrophages by T cells in RA mainly includes effects on macrophage activation, polarization, and osteoclast differentiation (**Figure 2**).

### T Cells Recruit Monocytes/Macrophages in RA

IL-17 from RA synovial fluid has a direct recruitment effect on monocytes *in vitro*. Moreover, human monocytes intravenously transplanted into SCID mice are recruited to ﻿implanted sponges pre-treated with human IL-17 (39). In this regard, tissue-immersed human Th17 cells secrete CCL20, which has chemotactic effects on monocytes (40). Nevertheless, this does not exclude the possibility that IL-17 may have indirect chemotactic effects on monocytes by inducing chemokine secretion from other cellular components of RA synovium. IL-17 in the ankle joint was associated with an increase of F4/80 (macrophage marker) and CCL2 levels. IL-17-mediated CCL2 upregulation involves PI3K, ERK, and JNK pathways.

However, not all T cells subtypes promote or activate the inflammatory status of macrophages in RA. In the presence of CD4^+^ CD25^+^ regulatory T cells (Tregs), primary human monocytes/macrophages survive while adopting an anti-inflammatory phenotype. ﻿The induction of monocyte death requires activation of CD4^+^ CD25^-^ responder T cell–cell contact in a FAS-L/FAS dependent manner (41).

### T Cells Promote Cytokine Production by Macrophages in RA

As early as 1994, Wagner et al. proved that plasma membranes from anti-CD3 activated human peripheral CD4^+^ T cells but not from resting CD4^+^ cells were able to activate monocytes to produce IL-1 in absence of co-stimulatory cytokines, in a CD40-CD40L dependent manner (42).

T cell receptor (TCR)/CD3-mediated T cell activation induces monocyte TNF-α production. It has been reported that addition of IFN-γ or GM-CSF to T cell and monocyte co-cultures enhanced T cell induction of TNF-α by monocytes from RA patients (43). Another study demonstrated the specific mechanism by which human T cells promote TNF-α secretion from macrophages: T cells pretreated with Rolipram or cAMP analogues inhibited the increase in proliferation induced by IL-15, expression of cell surface molecules CD69, LFA-1 and ICAM-1, and production of TNF-α from macrophages (44).

On the other hand, T cells can facilitate the production of anti-inflammatory cytokines from macrophages under other circumstances. IL-10 is an anti-inflammatory cytokine secreted in the joints of RA by macrophages and blood-infiltrating lymphocytes. It has been observed that IL-10 production in RA synovial-membrane mononuclear cells and M-CSF-primed macrophages is activated by interaction with ﻿cytokine-stimulated T cells in a PI3K- and p70S6K-dependent manner (45). However, the mentioned study did not explain which subtype of T cells promoted the production of anti-inflammatory cytokines by the macrophages.

### T Cells Regulate Osteoclast Differentiation in RA

The regulation of monocytes/macrophages by T cells in RA also reflects in the ability of T cells to regulate the differentiation of monocytes to osteoclasts, which is an important cause of bone erosion in RA patients (46). Bone absorption of osteoclasts leads to the production of “erosion points”, this has pathological significance in RA and can be used as an index of disease severity (47).

Miranda-Carús et al. found that T cells from peripheral blood of patients with early RA express the Receptor Activator for Nuclear Factor κ B Ligand (RANKL) and IL-15 on the cell surface, which promotes osteoclastogenesis of autologous monocytes; ﻿this process was inhibited by osteoprotegerin (OPG) and neutralizing monoclonal antibodies against IL-15, IL-17, TNF-α, and IL-1β (48). However, this study did not elucidate which T cell subtype induced osteoclast differentiation.

In a co-culture system, human IFNγ^+^ T cells promoted the M-CSF-induced differentiation of monocytes to osteoclasts through the expression of RANKL (49). However, IFNγ also disrupted the differentiation of murine osteoclasts *via* degrading RANK bridging protein TRAF6, this suggests that IFNγ^+^ T cells can promote or hinder osteoclastogenesis under different conditions (50). In this sense, Th17 cells are usually associated with osteoclastogenesis. Th17-related cytokines increase in RA synovium and directly induce osteoclast differentiation (51). In addition, murine RANKL^+^ Th17 cells have been demonstrated to change mature osteoclasts to the “bone absorption” status (52). It has been reported that T cells from synovial fluid of RA patients express high levels of RANKL and that high amounts of RANKL^+^ CD3^+^ T cells can be been found in synovial tissue of RA patients (53). Therefore, T cells found in RA can contribute to osteoclast formation, leading to consequent bone absorption.

Murine Th22 cells have been identified as a new subset of IL-22 producing cells (54). IL-22 production was considered characteristic of the CD3^+^ CD4^+^ CCR4^+^ CCR6^+^ CCR10^+^ cells, and as their ability to produce this cytokine exceeded that of other subgroups of Th Cells, the population was designated as Th22. It has been reported that co-culture of Th22 cells with monocytes in the presence of M-CSF and RANKL induced osteoclast formation more efficiently than Th1 cells or Th17 cells from RA patients (55). Overall, RA T cell-related cytokines could recruit, polarize, activate, or differentiate monocytes/macrophages. In RA synovium, the cellular phenotype of monocytes/macrophages may also depend on the synergic interaction between T cell-derived soluble factors and other cells.

## Mutual Interaction Between Macrophages and T Cells in RA

**Figure 3:**
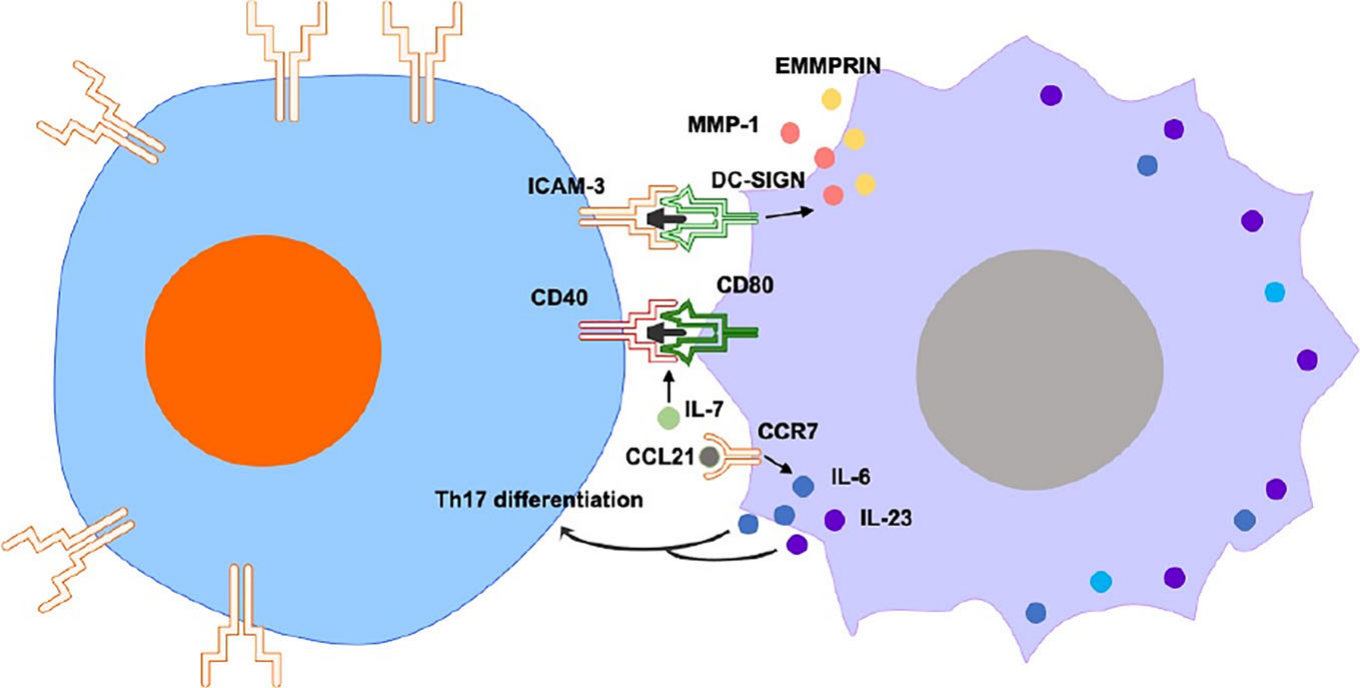
Mutual interaction between macrophages and T cells includes ICAM3/DC-SIGN, CD40/CD80 and CCL21/CCR7-Th17. The interaction of macrophages/T cells *via* DC-SIGN/ICAM-3 promotes the additional activation of synovial macrophages and production of EMMPRIN and MMP-1. IL-7 promotes co-stimulatory molecules CD80 and CD40 on CD14+ monocytes in the presence of CD4+ T cells. In the early stages of RA, CCL21-induced M1 cytokines favor the differentiation of naïve T cells into Th17 cells. In the erosive stages of RA, CCL21 aggravates RA osteoclastogenesis *via* M1 macrophages-mediated Th17 differentiation.

While certain studies focus on one-way regulation, other studies illustrate the mutual interaction between T cells and macrophages in RA (**Figure 3**). C-type lectin DC-SIGN is significantly expressed by CD68^+^ macrophages in synovium of RA patients. Expression of DC-SIGN and its ligand, intercellular adhesion molecule (ICAM-3, mostly expressed in T cells), is substantially detected in RA synovium, suggesting that the interaction of macrophages/T cells *via* DC-SIGN/ICAM-3 promotes the additional activation of synovial CD68^+^ macrophages and production of extracellular matrix metalloproteinase inducer (EMMPRIN) and MMP-1 (56).

CD4^+^ T cells and macrophages from RA synovial fluid were hyperresponsive to IL-7. This cytokine induced activation and proliferation of CD4^+^ T cells and monocytes/macrophages from synovium of RA patients in a cell contact-dependent manner. IL-7 also promoted co-stimulatory molecules CD80 and CD40 on CD14^+^ monocytes in the presence of CD4^+^ T cells (57). However, the specific molecular mechanisms by which IL-7 promotes activation of co-cultured T cells/macrophages remains elusive.

﻿In the early stages of RA, CCL21 treatment induced the ratio of M1-polarized macrophages, leading to up-regulation of IL-6 and IL-23 genes. These CCL21-induced M1 cytokines favor the differentiation of naïve T cells into Th17 cells. In the erosive stages of RA, CCL21 aggravated RA osteoclastogenesis *via* M1 macrophages-mediated Th17 differentiation. Consistent with the *in vitro* findings, an *in vivo* study showed that CCL21-mediated arthritis favors the exacerbation of joint inflammation into bone erosion, and that this process was associated with M1-macrophages dependent Th17 polarization. Therefore, CCL21 is an potential target for RA therapy, as the suppression of CCL21-mediated inflammation may relieve erosive arthritis modulated by the interaction of M1 macrophages and Th17 cells (58).

## The Effects of RA Therapies on T Cells and Macrophages

The imbalance of macrophages and T cell populations is an essential element to RA. Given the significance of T cell-monocyte/macrophage interactions in contributing to arthritis, targeting these interactions may be beneficial to treat inflammation related diseases. When we summarized the cytokines that mediate the crosstalk between macrophages and T cells in RA, TNF-α and IL-6 were found as two key cytokines that widely involved in the interaction between them. Currently, TNF-α and IL-6 related monoclonal antibodies are the effective targeted drugs for the treatment of RA. Therefore, people need to pay more attention to further clarify the cytokines-mediated interaction of macrophages and T cells in RA, which may help us to find more potential therapeutical targets of RA treatment.

In fact, uncovered mechanisms of existing therapies, such as CTLA4-Ig, may function by targeting monocytes/macrophages. For example, inhibition of IL-6 with monoclonal antibodies against IL-6R can increase the Treg ratio (59), but other mechanisms of action may include reducing the proportion of inflammatory monocytes, inducing monocyte apoptosis, and inhibiting IL-6 production in monocytes. The function of Treg cells was enhanced after treatment with TNF-α inhibitors (60). When antigen-presenting cells and CD4^+^ T cells are co-cultured, TNF-α blockage promotes the expression of IL-10 and immunomodulates effector CD4^+^ T cells. It has found that after TNF-α blockage, IL-17 and IL-10 are significantly induced in CD4^+^ T.

In addition, the JAK inhibitors, tofacitinib and ruxolitinib, have been shown to effectively suppress the inflammatory response of primary monocytes-induced macrophages from PBMCs preparations. Moreover, tofacitinib effectively inhibited the development of K/BxN serum transfer-induced arthritis models (STIA) (61). JAK inhibition can induce osteoclast differentiation. Furthermore, both tofacitinib and ruxolitinib were able to activate the feedback inhibition of IL-10-mediated transcription of cytokines, thereby blocking the production of LPS-induced cytokines in macrophages (62). Therefore, JAK inhibitors have the ability to regulate multiple cellular functions of monocyte/macrophage.

## Conclusion

Overall, there is emerging evidence that monocytes/macrophages and CD4^+^ T cells play a central role in RA. Except secretion of inflammatory cytokines, synovial monocytes/macrophages also produce chemokines that attract and maintain homeostasis of CD4^+^ T cells in synovium. Activated monocytes can affect the Th1/Th17 cells differentiation from CD4^+^ T cells. In addition, monocytes/macrophages affect the number and function of regulatory CD4^+^ T cells by producing certain cytokines. Similarly, CD4^+^ effector T cells can activate, polarize, and kill monocytes/macrophages and affect the chemotaxis of monocytes, while CD4^+^ Tregs can improve their survival and induce anti-inflammatory monocytes/macrophages. However, due to the number of studies on the interaction of these two kinds of cells in RA is limited, the interaction of T cell subpopulations and macrophages in RA is not fully investigated. Following studies should compare the worsening effects of pro-inflammatory CD4^+^ T cell subpopulations or monocytes/macrophages on anti-inflammatory monocytes/macrophages or T cell subpopulations and the ameliorative effects of anti-inflammatory T cell subpopulations or monocytes/macrophages on pro-inflammatory monocytes/macrophages or CD4^+^ T cell subpopulations, which can determine which immune cells play a more important “commander” role in joint synovium of RA.

Identifying additional cellular membrane markers able to reflect the subtype characteristics of monocytes/macrophages will help further investigate their specific role in RA. The specific role of subtypes of monocyte/macrophage is still elusive in animal models or immortal cell lines, the study of human primary monocytes and macrophages is essential to understanding the role of these cells in the pathogenesis of RA. Improving the knowledge on the ontogeny of synovial macrophages could help us achieve a deeper comprehension of the role of tissue-resident macrophages in synovium of RA. The direct interaction between CD4^+^ T cell subtypes and resident macrophages can further illustrate how the effector T cell response is produced *in situ* and how effector CD4^+^ T cells and Tregs differently regulate macrophages. A better understanding of how the interactions between these cellular components lead to immunopathology will facilitate the development of new treatment strategies and the improvement of the currently available strategies.

## Author Contributions

JT and WH drafted the manuscript. JT, TL, WZ, and CZ revised the manuscript. All authors contributed to the article and approved the submitted version.

## Funding

This work was supported by the National Natural Science Foundation of China (Grant No. 81871788 and 31900616), the Project for Science and Technology leader of Anhui Province (Grant No. 2018H177), the Scientific Research Fund of Anhui Education (Grant No. 2017jyxm1097), the Anhui Provincial Postdoctoral Science Foundation (Grant No. 2019B302), Youth Program of the Provincial Natural Science Foundation of Anhui (2008085MH247), and The project of improvement of scientific ability of Anhui Medical University(2020xkjT009).

## Conflict of Interest

The authors declare that the research was conducted in the absence of any commercial or financial relationships that could be construed as a potential conflict of interest.
